# IgG3 constant region GM (γ marker) allotypes could influence the neutralizing potency of anti–SARS-CoV-2 monoclonal antibodies

**DOI:** 10.1073/pnas.2119380119

**Published:** 2022-02-01

**Authors:** Janardan P. Pandey

**Affiliations:** ^a^Department of Microbiology and Immunology, Medical University of South Carolina, Charleston, SC 29425

In their timely and well-designed study, Kallolimath et al. (1) report up to 50-fold increased neutralization potency of an anti–severe acute respiratory syndrome coronavirus 2 (anti–SARS-CoV-2) monoclonal IgG3 antibody, compared to antibodies of the other three IgG subclasses with an identical antigen-binding site. They attribute this heightened potency to the long hinge region of IgG3, facilitating the cross-linking of the spike protein on the viral surface. I would like to suggest an additional explanation for this interesting observation, based on the extensive genetic polymorphism of the Fc region of IgG3, and several lines of evidence documenting the contribution of the constant region of antibodies to the expression of variable region epitopes and their interaction with antigen (2–4).

Among IgG subclasses, IgG3 is the most polymorphic, with 13 GM alleles, compared to 4 for IgG1 and 2 for IgG2. As first suggested by J. B. S. Haldane, major infectious diseases have been the principal selective forces of natural selection (5). So, the extensive polymorphism of IgG3 may be a result of the selective pressure exerted by infectious diseases during our evolutionary history. Association of IgG3 allotypes with immunity to several infectious pathogens supports this contention (6). The IgG3 antibody described by Kallolimath et al. (1) expresses the GM5 allotype. It is possible that the amino acid substitutions responsible for the expression of the serologically detectable GM5 allotype cause conformational changes in the antigen-binding variable region which increases their neutralizing potential. Contribution of both variable and constant regions to the expression of certain antigen-binding region idiotypes has been known for several decades (3). More recent studies have provided a large body of evidence documenting the contribution of the constant region to the thermodynamic parameters of antibody variable regions (7).

I concur with the authors (1) regarding “the need for a better understanding of the molecular properties of SARS-Cov-2 Ab variants prior to application in therapy or prophylaxis.” Therefore, I suggest manufacturing 13 versions of anti–SARS-CoV-2 IgG3 antibodies, each expressing a different GM allotype. One of these variants might be even more potent in its neutralizing efficiency than the GM5-expressing antibody engineered by the authors.
